# Reduced short term adaptation to robot generated dynamic environment in children affected by Cerebral Palsy

**DOI:** 10.1186/1743-0003-8-28

**Published:** 2011-05-21

**Authors:** Lorenzo Masia, Flaminia Frascarelli, Pietro Morasso, Giuseppe Di Rosa, Maurizio Petrarca, Enrico Castelli, Paolo Cappa

**Affiliations:** 1 Robotics Brain and Cognitive Sciences Dept., Italian Institute of Technology (IIT), Genoa, Italy; 2 Pediatric Neurorehabilitation Division, IRCCS Children Hospital 'Bambino Gesù', Palidoro Rome, Italy; 3 Mechanics and aeronautics Dept., 'Sapienza' University of Rome, Rome, Italy; 4 Dept. of Informatics, Systems and Telematics, University of Genoa, Italy; 5 Physical Medicine and Rehabilitation, 'Sapienza' University of Rome, Rome, Italy

## Abstract

**Background:**

It is known that healthy adults can quickly adapt to a novel dynamic environment, generated by a robotic manipulandum as a structured disturbing force field. We suggest that it may be of clinical interest to evaluate to which extent this kind of motor learning capability is impaired in children affected by cerebal palsy.

**Methods:**

We adapted the protocol already used with adults, which employs a velocity dependant viscous field, and compared the performance of a group of subjects affected by Cerebral Palsy (CP group, 7 subjects) with a Control group of unimpaired age-matched children. The protocol included a familiarization phase (FA), during which no force was applied, a force field adaptation phase (CF), and a wash-out phase (WO) in which the field was removed. During the CF phase the field was shut down in a number of randomly selected "catch" trials, which were used in order to evaluate the "learning index" for each single subject and the two groups. Lateral deviation, speed and acceleration peaks and average speed were evaluated for each trajectory; a directional analysis was performed in order to inspect the role of the limb's inertial anisotropy in the different experimental phases.

**Results:**

During the FA phase the movements of the CP subjects were more curved, displaying greater and variable directional error; over the course of the CF phase both groups showed a decreasing trend in the lateral error and an after-effect at the beginning of the wash-out, but the CP group had a non significant adaptation rate and a lower learning index, suggesting that CP subjects have reduced ability to learn to compensate external force. Moreover, a directional analysis of trajectories confirms that the control group is able to better predict the force field by tuning the kinematic features of the movements along different directions in order to account for the inertial anisotropy of arm.

**Conclusions:**

Spatial abnormalities in children affected by cerebral palsy may be related not only to disturbance in motor control signals generating weakness and spasticity, but also to an inefficient control strategy which is not based on a robust knowledge of the dynamical features of their upper limb. This lack of information could be related to the congenital nature of the brain damage and may contribute to a better delineation of therapeutic intervention.

## Background

Cerebral palsy (CP) is a group of non progressive, but often changing, motor impairment syndromes secondary to lesions or anomalies of the brain arising in the early stages of development [1,2]. Although motor impairment is the leading factor in CP, sensory disorders have been described [3] and sensorimotor cognitive functions are probably affected due to the complexity of the motor impairments implying primary and secondary deficits [4-6].

The last two decades has brought a tremendous depth of understanding to the function of central nervous system (CNS); if on one side molecular biology applied to neurogenetics provided unprecedented insight into the pathologic mechanisms of neurologic disorders, on the other side the use of robotics, as a non-invasive investigating tool of motor recovery and rehabilitation, offered the possibility to accurately observe and quantify human movements as the result of the response of the CNS to an external dynamic interaction. In the field of motor control many paradigms were proposed to study the sensorimotor adaptation to force field. Since the seminal work of Shadmehr and Mussa-Ivaldi [7], multijoint reaching movements under application of robot generated deviating forces have been used in order to understand how the CNS controls movements in dynamic conditions: it was found that healthy subjects are able to gradually tune an internal model of the arm-environment which allows to perform almost straight trajectories, thus compensating the effect of the force field. A key finding of motor adaptation is that when the external force is unexpectedly removed the subjects move as if the force was still active, making error in the opposite direction to the one of the generated force. This motor *after-effect *[8] demonstrates that the reaction to an external structured dynamics is the result of an *anticipatory strategy *[9,10] gradually developed by the CNS while experiencing the robot altered dynamic. Adaptation and its related *after-effects *have been demonstrated for a variety of structured force fields, dependent on different kinematic/dynamic features of the movements: position [11], acceleration [12], Coriolis force [13], velocity [14], and skew symmetric "curl" fields [15].

Patton et al. [16] used deviating force field in adult stroke subjects as *error-enhancing *robot therapy, and it was demonstrated that impaired subjects are still able to learn the force field and the rehabilitation approach was even more effective than the assistive (*error-reducing*) one.

The studies on healthy subjects allowed to shed some light on an another important phenomenon: the magnitude of kinematic error varies as subjects move in different directions while experiencing the same structured force field [17,18]. A computational analysis of the problem suggested that the directional differences in kinematic error may arise from spatial asymmetries in arm impedance [19]. Darainy et al. [20] have shown how in healthy subjects the anisotropic features of the arm impedance play a fundamental role in motor learning and generalization. In general terms an important concept in motor control is the idea that the CNS uses an internal model of the motor system and of the surrounding environment to predict the sensory consequences of commands [21]. In healthy subjects this model allows to account for the inertial anisotropy of the arm and consequently generate the right motor commands to counteract the external forces. However in impaired subjects, although able to adapt [22-26], it is not clear whether the CNS maintains an intact capability of predicting the arm dynamics and compensate for the anisotropy or not.

The brain injury resulting in cerebral palsy (CP) occurs early in neurodevelopment or birth accident, whereas stroke occurs generally in adult life. There are many scientific results which suggest that plasticity is greater in the developing brain than in the mature one [27,28]. Despite evidences that are observable in adults, the ability of CP subjects to deal with the central planning issues associated with control of arm is still an open question. The recovery mechanisms in children are quite different from adults [29,30], due to their higher plasticity and because the limbs control ability is age-related. The goals of the current study were twofold: 1) to ascertain if impaired children affected by CP preserve the ability to adapt to a force field; 2) to investigate which differences in kinematics between CP and Control groups lead to an unequal learning rate. We believe that these findings may suggest new therapies, as it has been demonstrated in healthy adult stroke patients [31-33]. Moreover we agree with Papavasiliou [2] that the introduction of new therapies facilitates an individualized management plan and multimodal treatment is optimized with a multidisciplinary team.

## Methods

### Subjects

Fourteen subjects volunteered to participate to the experiment (table 1): 7 CP pediatric subjects, mean age 10.14 years (range 7-14) recruited at the Neurorehabilitation Division of the Pediatric Hospital Bambino Gesù (Rome, Italy) and 7 age-matched right-handed healthy control subjects (mean age 9 years, range 8-14). Research was approved by the ethical committee of the Hospital and conforms to the ethical standards laid down in the 1964 Declaration of Helsinki. Before starting the protocol, the parents were asked to sign a consent form. All CP subjects were in the chronic stage and were affected by hemiparesis with a moderately impaired upper limb function. The table also reports the score for three relevant clinical scale: 1) the elbow modified Ashworth scale of spasticity, 2) the arm section of the Fugl Meyer scale, and 3) the Melbourne scale. The subjects of the CP group were also characterized by the following exclusion criteria: 1) bilateral impairment, 2) severe sensory deficit of the impaired limb, 3) linguistic-cognitive impairment at a level that would not allow to understand the task and perform the experiment, 4) use of drugs, as botulin toxin therapy, that would affect muscular properties.

**Table 1:** Patients demographics (CP group)

CP GROUP	Age	Gender	Pathology	Brain lesion	Affected hand	Elbow modified Ashworth	Fugl_Meyer	Melbourne
**S1**	**7**	**Male**	**CP**	Left frontal cortex	**R**	**1**	**45**	**80**

**S2**	**11**	**Male**	**CP**	Left frontal cortex	**R**	**1+**	**43**	**77,8**

**S3**	**10**	**Male**	**CP**	basal ganglia bilaterally	**R**	**2**	**30**	**55**

**S4**	**7**	**Male**	**CP**	Left frontal cortex	**R**	**1+**	**41**	**73,7**

**S5**	**8**	**Male**	**CP**	Left middle cerebral artery	**R**	**1**	**42**	**76,7**

**S6**	**14**	**Male**	**CP**	Left pons and internal capsule	**R**	**2**	**45**	**74,7**

**S7**	**14**	**Male**	**CP**	Left subcortical	**R**	**1**	**42**	**68,8**

### Experimental apparatus

**Figure 1:**
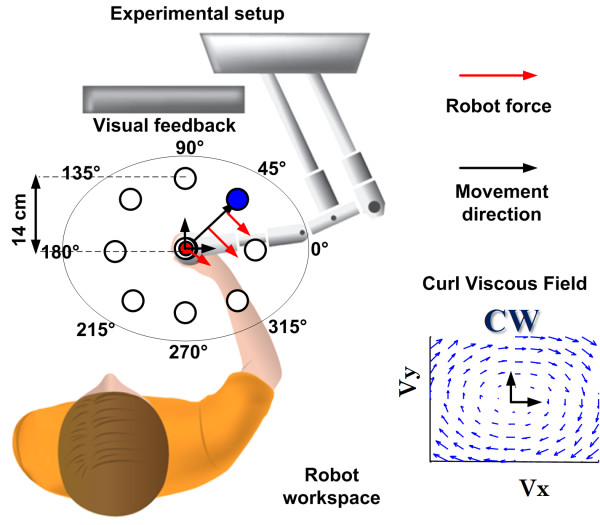
**Experimental setup**: the elliptical workspace of the robot is included in the reachable workspace of the subject. The shoulder of the subject is aligned with the centre of the workspace of the robot. The subjects were pre-tested in order to check if they were able to reach all the targets presented during the experiment. A clockwise (CW) curl viscous field was used.

The subjects grasped a planar manipulandum specifically designed for rehabilitation and evaluation of motor control: Inmotion2 Robot (Interactive Motion Technologies Inc., Boston, MA, USA). The robot is equipped with absolute encoders, which acquire hand/handle position with a 100 μm accuracy, and with direct-drive motors, which can transmit to the hand of the subjects force vectors with controllable amplitude and direction. In addition to the hand trajectories, determined by the interplay of the robot-generated force vectors and the subject-generated motor commands, we also computed the time course of the velocity and acceleration of the hand, as well as the corresponding jerk signal that is considered an indicator of smoothness of the trajectory formation process. Subjects were comfortably seated so that the center of the manipulandum workspace was approximately coincident with the center of their reachable workspace (see figure 1). Trunk strategy compensation was prevented by means of a seat belt; the elbow was supported in the horizontal plane by an anatomical orthosis; wrist motion was constrained by a thermoplastic cast in order to avoid a compensatory strategy of the wrist [34].

### Task

Subjects were instructed to perform reaching movements from the centre of the workspace to one of eight peripheral targets and back; each movement has to be performed in a specified time range of 1.2 ± 0.3 seconds. The peripheral targets were distributed uniformly (with a 45 deg spacing) on a circle with a 14 cm radius. A target-set (*TS*) consisted of 8 center-out movements plus 8 return movements. The sequence of center-out movements was randomized. Target and hand positions were presented as circles on a computer screen (visual feedback). The criterion for target reaching was a positioning error less than 5 mm and residual oscillations of the hand with speed smaller than 1 cm/s. When the criterion was met there was an acoustic feedback and the next target was shown. The robot was also used in order to perturb the movements of subjects and to evaluate motor adaptation. The perturbations were characterized by a Curly Viscous Field, i.e. a pattern of force vectors with amplitude proportional to the instantaneous speed of the hand and a direction perpendicular to the corresponding direction of the velocity vector:(1)

*F *is the force vector applied by the robot to the handle; *x*_*hand *_is the position vector of the hand; *λ *is the parameter of the force field. For this parameter we choose the following value *λ *= 20 Ns/m. This means that when the hand reaches a velocity of 1 m/s (typically the peak velocity of a reach movement) the force generated by the robot, which pushes the hand laterally, has an amplitude of 20 N. As shown in figure 1, the force field rotates in the clockwise direction. The field rotational direction would be inverted by changing the sign of the *λ *parameter. The value of *λ *= 20 Ns/m was tuned after a pilot test in order to provide a sufficient deviating force even in case of reduced speed of the subject's hand.

### Protocol

The protocol included a total of 640 pointing movements, distributed in 40 target sets (*TS*s), and was broken down into the following experimental phases:

- *Familiarization phase (FA)*: it consisted of 10 *TS*s, equivalent to 160 center-out movements. The main purpose was to allow the subjects to experience the inertial load of the robot, which is not negligible, in spite of the fact that the robot is highly backdriveable.

- *Curl Viscous Field Adaptation phase (CF)*: it included 20 *TS*s (i.e. a total of 320 movements) with exposure to a curl viscous field described by equation 1. During this phase the force field was randomly switched off five times for each direction (40 *catch trials *total).

- *Wash out phase (WO)*: 10 *TS*s (for a total of 160 center-out movements) without disturbing force, as in the FA phase.

CP subjects were encouraged to carry out the pointing movements to the target with an average velocity comparable to the control subjects, in order to ensure that the two groups experienced a similar level of force during the *CF *phase.

### Data analysis

The two cartesian components of the pointing trajectories were sampled at 200 Hz and smoothed by using a 6th order Savitzky-Golay filter, with a 170 ms window (cut-off frequency: ~11 Hz). The same filter was also used to estimate the time derivatives of the trajectory. *Movement onset *and *Movement termination *were then evaluated, by detecting when the hand speed curve crosses a suitable threshold value (0.05 m/s), in order to isolate each individual reaching movement. The following indicators were extracted from the recorded data for each targeting movement:

- *Lateral deviation (LD)*: it is defined as the deviation from the straight line that connects the initial position to the target, evaluated at the time of peak velocity. Positive and negative errors correspond to leftward and rightward lateral deviations, respectively.

- *Acceleration peak*: it is the highest value of the acceleration profile. This indicator, if associated with movement direction, can provide a polar plot that we expect to be asymmetric, as suggested in a previous study [21] that analyzes the anisotropy of the inertial properties of both the human and robot arms.

- *Peak and average speed*: as previously noted, these indicators were monitored for verifying the substantial equivalence of the field intensity in the two groups of subjects (CP and Control).

- *Directional analysis*: it was performed for each of the previously defined variables, in order to highlight the interplay between the effect of the deviating force field and the effect of the anisotropy of the mechanical impedance. We expect indeed that the CP group may differently control the inertial anisotropy of the arm while performing reaching movements. We also defined an "anisotropy index" *E *which measures the degree of "roundness" of the directional interpolating ellipses, by considering the major and minor semi axes of the ellipse (*a *and *b*, respectively):(2)

E = 0/1 for a perfectly round or completely flat ellipse, respectively.

- *Learning Index*: the degree of adaptation to the force field was measured by means of the following formula [35] that takes into account the values of lateral deviations in the force-field and catch trials (*LI*_*ff *_and *LI*_*catch *_respectively):(3)

_*LI *_ranges from 0.0 (null adaptation) to 1.0 (complete adaptation).

We assessed adaptation using a repeated measure ANOVA with three factors: group (CP vs. Control), phases (FA vs. FF vs. WO) and time (early vs. late phase). During the statistical analysis of the results, all hypotheses were tested using a significance level of 0.05.

## Results

**Figure 2:**
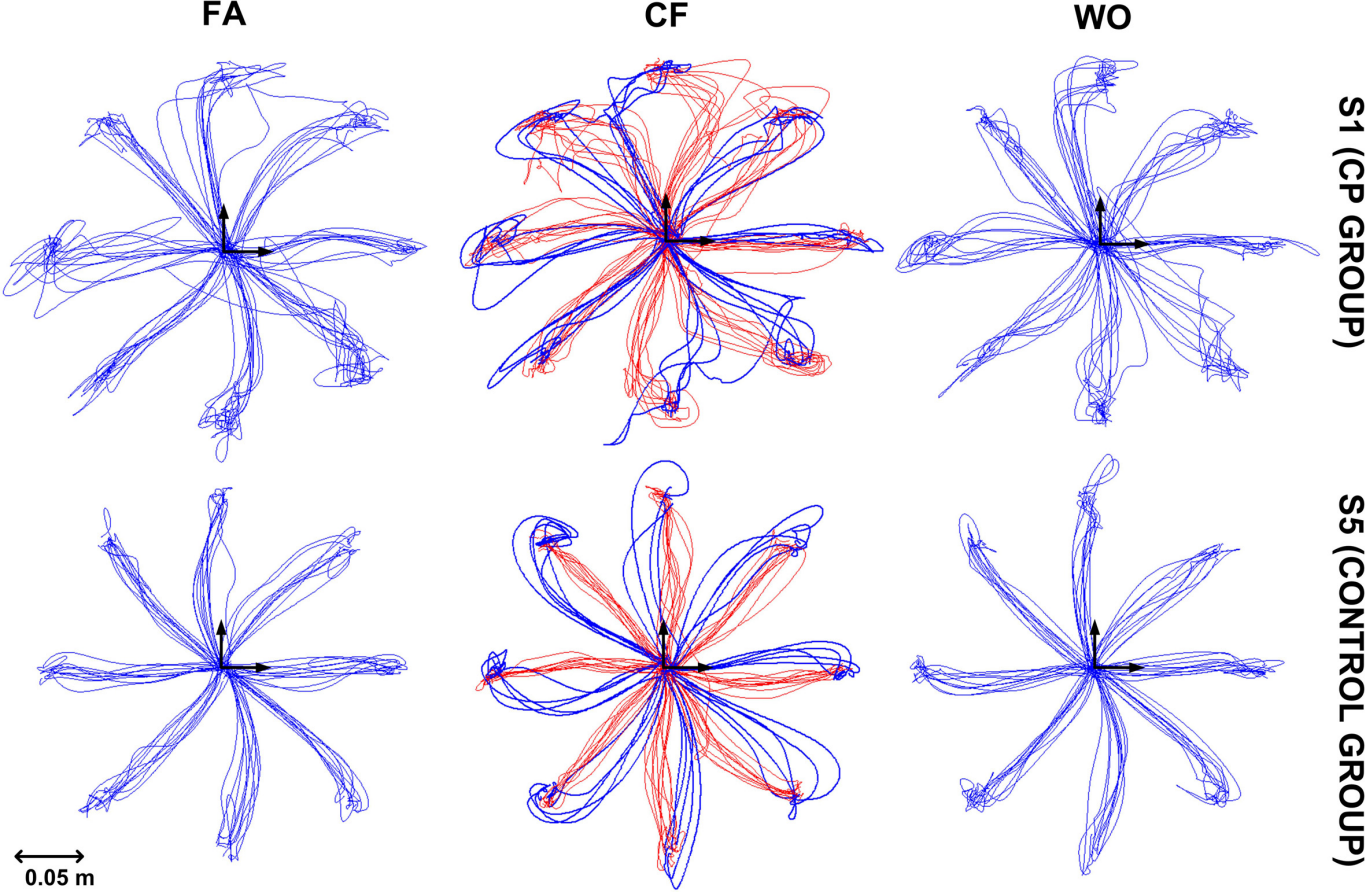
**Trajectories during the different experimental phases for two representative subjects, from the CP and Control group**. The blue traces correspond to movements with no disturbing field: all trials in the Familiarization phase (FA) and Wash-Out (WO) phase and catch trials in the Curl Viscous Field Adaptation phase (CF). The red traces correspond to movements affected by the disturbing force during the CF phase.

All the subjects were able to perform the assigned task in the different experimental phases. Moreover, we found that the control subjects and the CP subjects present similarities in moving their hands in the force field. Figure 2 shows the trajectories (form the center to the peripheral targets) during the entire experiment for two representative subjects form the CP and control group. For both subjects the effects to the force field are evident (CF phase, red trajectories), although the unimpaired subject learns to compensate the force in a more effective manner performing straighter trajectories. Catch trials in the CF phase (blue lines) reveal the presence of an anticipatory strategy and after effects in the WO phase indicates the compensation activity of the force field continues after the robot dynamics is removed.

The subject 1 (S1) from the CP group is mildly impaired, in fact during the FA phase his trajectories appear to be comparable with the ones of the unimpaired subject. An accentuation of lateral error in the FA phase is anyway visible especially in some directions (90°N, 270°S, 45°NE) corresponding to those in which the inertial effect of the arm coupled with the robotic device is higher. The same effects along the same target directions are also observable during the CF phase where the action of the deviating field is in fact less compensated and also the catch trials seem to be characterized by a higher lateral deviation.

**Figure 3:**
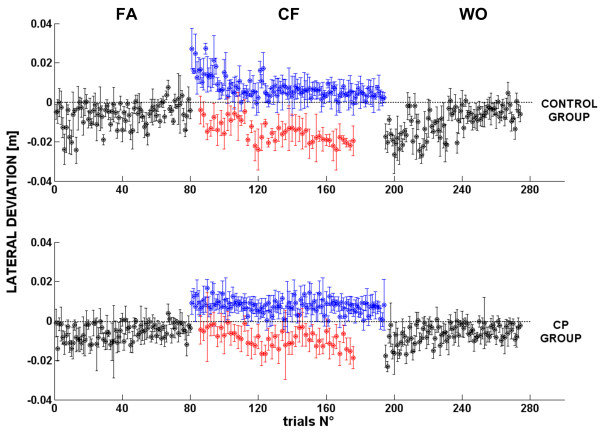
**Trend of adaptation for the CP group (bottom) and control group (top) over the different target-sets**; it refers to all the trials where black dots are average values of lateral deviation for all the subjects during familiarization and wash-out, while blue and red are referred to lateral deviation during force field adaptation and catch trials respectively. Error bars for each value refer to standard error over all subjects.

Let us consider first the subjects of the control group (upper panel of figure 3). Namely, they perform quasi straight trajectories during the FA phase; the trajectories are bent in the direction of the force field in the initial part of the CF phase, as a consequence of the external disturbance, but the subjects quickly learn to compensate reducing the lateral deviation by the end of the CF phase; the feedforward nature of the compensation mechanism is proved by the catch trials, which show a bending (lateral error) in the opposite direction; at the end of the beginning of the WO phase an *after effect *is clearly noticeable and the original performance is restored at the end of the WO.

Contrarily the CP subjects (lower panel of figure 3) clearly perform in a less reliable way, i.e. with a larger variability, but still they can carry out the task of reaching the targets in approximately the prescribed time, in all the directions and in the different phases.

**Figure 4:**
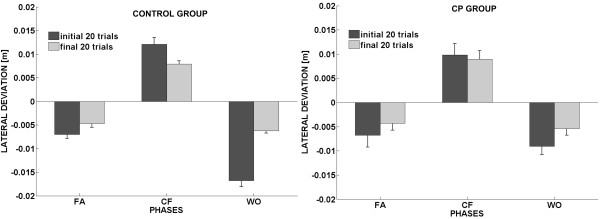
**Early Vs Late performance of the two groups**. Bar plots of the early (initial 20 trials) and late (final 20 trails) exposition the experiment in the three experimental phases, with standard error for each bar.

A further insight of the learning capability of the two groups is provided by the bar plots of the early and late training in the two groups: figure 4 depicts the comparison between the average values of the initial and final twenty trials in the different phases of the experiment. Observing the performance of the two groups during the different phases of the experiment we noticed that they both show a statistical significant reduction of the lateral error between the early and late phase of the familiarization [F(1,12) = 8.2506, p = 0.01402] and early and late phase of the wash-out [F(1,12) = 31.239, p = 0.00012].

It is anyway crucial to precise that the experiment was mainly focused on testing if CP subjects were able to develop an anticipatory strategy to counteract a deviating force field. Nevertheless, in terms of trajectory corrections it is clear from figure 4 that CP group does not succeed in significantly decreasing the lateral deviation over the course of the trials; as depicted in the CF phase of figure 4 the early and late value of the lateral deviation do not show any significant difference [F(1,12) = 0.04, p = 0.8384]. Contrarily, control subjects show a distinct change between the initial and the final part of force field exposition [F(1,12) = 6.61, p = 0.00051].

In order to evaluate the degree of motor adaptation it is necessary to inspect the *after-effects*. When the disturbing force field is removed at the beginning of the WO phase, both groups show lateral errors in the opposite direction of the force field. This error magnitude provides a measure of how much the subjects developed an anticipatory strategy of the robot generated dynamics over the course of the exposition to the field. There is a statistical significance decrease of the lateral error between late familiarization and early wash-out [F(1,12) = 23.5999, p = 0.00039] and also interaction between the two main effects phases and groups [F(1,12) = 7.2208, p = 0.01977] showing a lower *after effect *of the CP group than the one present in control group. The latter seems to have a better adaptation rate to the force field, between the early and late values of the lateral deviation, than the CP group, exhibiting a greater after-effect and indicating that they are more prone to learn the force field and compensate the deviation with a lower lateral error at the end of the training.

**Figure 5:**
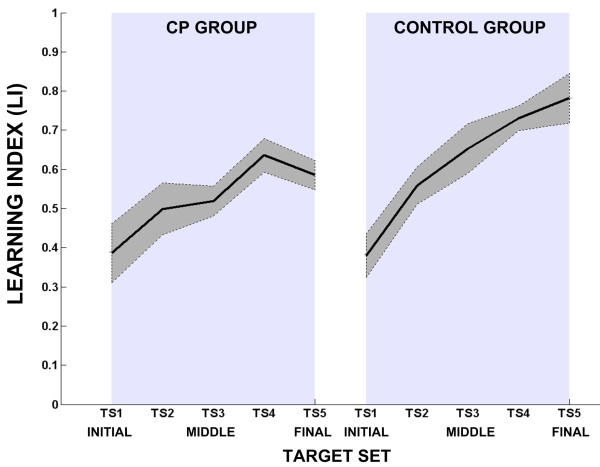
**Learning index for CP and Control groups for 5 different target sets (TS)**. Each learning index value is computed as the average of eight catch trials, taking into account the corresponding directions, when the force field is active in the CF phase. Solid lines indicate mean value and dashed lines indicate standard error.

Moreover, a strong indication of the reduced short-term adaptation of the CPs is clearly shown by the learning index (LI) depicted in figure 5. The LI corrects for possible difference in performance due to differences in the action of the force field; if adaptation occurs, during force field the lateral deviation decreases while its value increases for the trajectories performed during catch trials due to the higher compensatory action by the CNS. Figure 5 shows that in the control group LI grows monotonically, in the initial learning, and this behavior is followed by an exponential trend, as expected for a short term adaptation experiment. In contrast, in the CP group LI is characterized by a lower increasing trend, with an early saturation, which implies a reduced learning capability.

**Figure 6:**
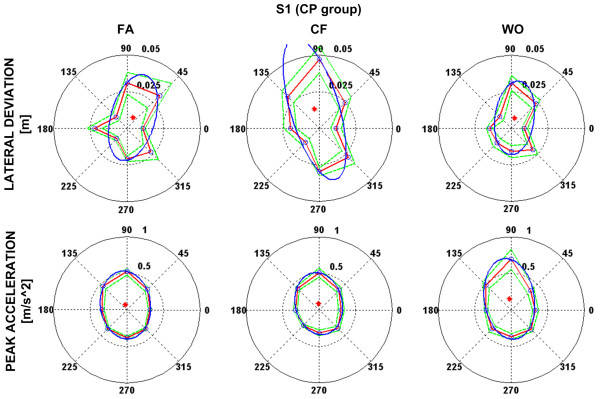
**Polar plot of lateral deviation (top) and peak of acceleration (bottom) for a representative subject (subj_1) from CP group**. Red lines along the different directions are the average of the lateral deviation and peak acceleration values. Green lines represent the standard error and red dot is the centre of the interpolating ellipse. The blue ellipse is an interpolation of the average values over the different directions.

Movement anisotropy of the human arm is responsible for directional variability of movement kinematics [36], therefore a directional distribution of acceleration peaks while moving along the targets may help in comprehending how inertial anisotropy plays an important role in the adaptation performance to the robot generated force field. Figure 6 (referred to S1 of the CP group) shows the polar plots of the lateral deviation and the acceleration peak, i.e. the distribution along the different target directions of the two indicators. As shown in figure 6 the kinematic error is distributed towards those geometrical configurations in which the arm and the robot together may result more difficult to control by the subject. Observing the average of acceleration peaks along the different directions (bottom figure 6), one can realize how the orientation of the major axes of the interpolating ellipses seems to be concordant with the ones interpolating the lateral deviations, over the three different phases of the experiment; we may hypothesize that the higher is the lateral deviation over a certain direction and the higher is the correspondent acceleration peak due to the trajectory correction operated by the subject. The directional distribution of the kinematic variables seems to shed some light on how the subjects try to master the interaction with the generated external environment by taking into account the anisotropy of the arm.

**Figure 7:**
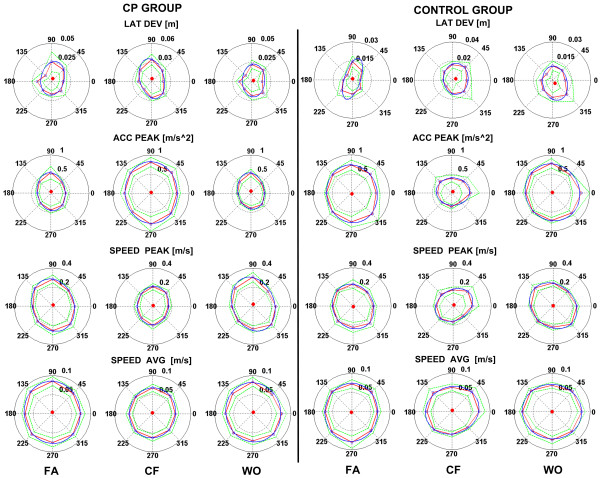
**Polar plot of the different performance indicators during the different phases**. (FA: familiarization; CF: curl field adaptation; WO: wash-out) for the two CP and control groups 1) Lateral deviation (first row of panels), 2) Acceleration peak (second row), 3) Speed peak (third row), 4) Average speed (fourth row). The red, continuous lines link the average values and the green lines link the corresponding standard errors. The ellipses are derived from an interpolation of the average values and the red point represents its center.

Under this hypothesis it might be useful to observe the directional distribution of the acceleration and speed for both the CP and control groups. Figure 7 shows the polar plots of the lateral deviation, acceleration peak, velocity peak and average velocity, respectively, averaged for the two groups, during the three experimental phases (FA, CF, WO). Observing the mean speed (bottom of Figure 7) it appears that the values of the linear velocity are comparable for both CP and control subjects and therefore the curly viscous field is perceived by the two groups with a similar amount of deviating force.

However, the inspection of the polar plots of the lateral deviation, acceleration and speed peaks depicts different behaviors of the two groups. In order to analyze and quantify the differences among these kinematic variables, we computed an eccentricity index of the interpolating ellipses according to the following formula:(4)

where ***b ***and ***a ***are the minor and major semi axes of the interpolating ellipses. This measure elucidates how kinematic and the dynamic features of the movements are directionally varied in response to the application of the force field.

**Figure 8:**
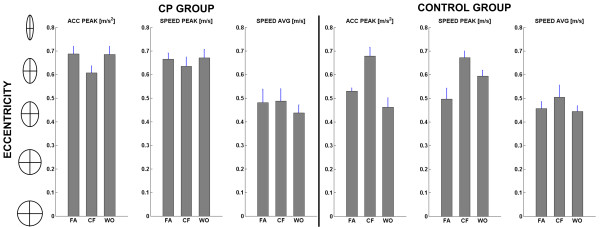
**Degree of anisotropy of the interpolating ellipses**. The eccentricity was evaluated for the acceleration peak, velocity peak, and average velocity, after interpolation of the CP and Control group in the three experimental phases (FA, CF, WO).

Figure 8 shows that the eccentricity of the interpolating ellipses for control group dramatically increases during the CF phase with respect to the FA and WO phases for acceleration peaks and speed peaks [F(2,18) = 11.89, p < 0.001; F(2,18) = 6.78; p = 0.006]. It means that unimpaired subjects in order to balance the force field are able to re-compute and orient the directional distribution of the acceleration and velocity, leading to a better performance while reaching the different targets: the change in the kinematics over the eight directions, suggests that the force field is perceived as a disturbance and in order to perform straight trajectories subjects are forced to directionally tune the control strategy of the arm. Observing Figure 8, it is evident that contrarily CP groups, when the force field is active, do not adjust the kinematics of the movements; in fact the values of the eccentricity, in the three phases of the experiment, don't differ for acceleration and speed peaks [F(2,18) = 2.21; p = 0.1385; F(2,18) = 0.48; p = 0.6264].

## Discussion

It is widely accepted from previous studies [37,38] that the generation of coordinated multijoint movement requires the CNS to account for joint interaction effects by relying on suitable "internal models" of the intrinsic arm dynamics. It is suggested that such models are acquired by motor learning and are used by the CNS in order to provide feed-forward control commands, capable to compensate for the anisotropy of the arm and the corresponding dynamics [39]. Moreover, haptic robots have been used for exposing human subjects to novel, extrinsic dynamic environments [40-43] whose effect is added to the effect of the intrinsic dynamics. In this way it was possible to investigate the compensatory strategies adopted for short term adaptation. It appears that adaptation involves changes in the cerebellar cortex [44,45], suggesting that internal models of the external dynamics are stored in the cerebellum [46,47]. It is also hypothesized that the ability of controlling our limbs is acquired early in life and is then continuously updated in order to accommodate gradual biomechanical, muscular, and neural changes that occur during development and above all in childhood.

As pointed out by Shadmehr et al [48] two main problems must be solved by the brain for the acquisition of efficient internal models: 1) sensory feedback is noisy and delayed, making movements inaccurate and potentially unstable; 2) the causal relationship between motor commands and ensuing movement is somehow unpredictable, as the body/environment dynamics is ceaselessly changing. Forward internal models of the body/world ensemble can solve such problems by providing predictions of the state of the body as it interacts with the world around it. However, such models are only useful if they produce unbiased predictions and this requires, at the same time, that the level of noise in the system is sufficiently low and the sensorimotor system is well calibrated.

Another important aspect that must be considered is that human motor development is the result from a complex interaction between gene and experience, and the somatopic organization of the primate motor cortex (M1) emerges postnatally. As suggested by Stoeckel et al. [49] an altered motor experience during early motor development may play a more critical role in the shaping of genetically determined neural networks underlying control of movement.

The present study demonstrates that both groups of subjects completed the required task without difficulties, although there is evidence of different performance between the impaired and unimpaired subjects. In spite of their young age, which implies shorter arms and smaller weights than the adults recruited in classical studies of force field adaptation, the control group behaves in a very similar way. Therefore, these subject are capable not only to learn a compensation strategy of the disturbance (equivalent to the one developed by their adult counterparts) but also to predict the non-linear dynamics of the robot which, in relative terms with respect to the intrinsic arm dynamics, is much more relevant for the children than the adults.

However there are differences between the two groups as regards control and adaptation capabilities. The subjects should learn how to master the combined dynamics of the robot and the arm and the inspection of figure 3-4 suggests that the strategy acquired by control subjects may be more efficient that the one developed by CPs, having a faster decrease rate of the lateral error in the CF phase; moreover, during the catch trials (red dots in the CF phase for the CP group) there is a tendency to increase the value of the lateral deviation as a result of the increasing compensatory force exerted by the subjects over the course of the experiment when the force field was unexpectedly removed. After training, an after-effect is also visible in the initial trials of the WO phase, where the kinematic errors appear to be shifted and opposite with respect to those in last trials of the CF phase.

In this context, the aim of the present study was to understand to which extent children affected by congenital hemiparesis have a reduced ability to acquire a predictive force field compensation strategy because of a lack of an efficient feed-forward control mechanism. A crucial point is then the meaning of the *after-affects *and catch trials observed during the experiment: is there evidence of anticipatory mechanisms of adaptation, although degraded, or do CP subjects merely react to the robot generated force without anticipating sensory commands? An answer to the question may be found by looking at the inability of CP subjects to master the arm anisotropy and to use it depending of the external dynamics. As reported in previous paper by Gordon et al. [35] the early portion of hand movement is characterized in term of spatial distribution of the acceleration, that for healthy subjects result in a systematic directional variation. This phenomenon can be explained as an inaccurate account of the inertial anisotropy of the arm, persisting in adults even when robust information of the arm is already developed. Previous studies [50] demonstrated how healthy children adapt to robot generated force field, but performance is still more variable than adults, due to movement inconsistency and not motor adaptation inability. This outcome suggested that higher movement variability in young children may arise from higher motor noise and constraining physiological factors of the developing motor system; in fact computational processes taking part to internal model formation are implemented by the CNS early in development and they need to account for continual control adjustment in order to compensate for morphological growth during the development.

We can hypothesize that in children this updating process, associated to a higher motor noise, plays a leading part on motor learning especially in those cases even weakened due to cerebral palsy, and thus the inertial anisotropy during movement may strongly influence subjects' ability in mastering complex interaction with the surrounding environment.

We believe these spatial abnormalities in CP children result from a systematic disturbance in the motor control signals to be attributed not only to weakness and spasticity [51] but above all to a deficient control strategy based on a robust knowledge of their arm dynamics. It is conceivable indeed that an indirect effect of weakness and spasticity is to degrade the capability of the brain to calibrate the sensorimotor system, thus making impossible the acquisition of a reliable internal model of prediction.

Although in the CF and WO phases the two groups exhibit comparable trends for catch-trials and after-effects, it is hard to demonstrate, for the CP group, that they are due to an anticipatory control strategy acquired during the exposition to the force field. After effects and catch trials may indeed be primarily related to a different interpretation of the CP subject of the force generated by the robot rather than a learning effect due to sensorimotor adaptation. In fact, control subjects interacting with the robot are able indeed to adapt to the force field while changing the directional distribution of the arm dynamics and kinematics as shown in figure 7 and 8; in contrast, CP children exhibit high lateral deviation and non-significant difference between the early and late phase of the short term adaptation, but more important they don't readjust directionally the dynamics of their movements as confirmed but the invariant orientation of the directional ellipse over the course of the experiment.

But why children affected by cerebral palsy, even at a mild level, do not show same performance as unimpaired subjects? A possible answer may come from the distinction proposed by Huang and Krakauer [52] between *motor adaptation *and *skill learning*. From a control point of view, *skill learning *can be seen as a global control scheme to solve the task, while *motor adaptation *is the tuning of the skill learning in order to compensate a change in the operating condition (i.e. change in environment dynamics). Is the velocity of tuning and therefore the adaptation rate between impaired and healthy subjects different? Scheidt and Stoeckmann [53] compared force field adaptation in post-stroke and healthy subjects. It was found the two groups used the same compensatory strategy but the influence of the successive trials was lower in stroke subjects, indicating that impaired people (stroke) preserve adaptation capacity as healthy subjects but they require more practice. In our experiment short term adaptation was tested for CP children and control unimpaired group; as expected the results demonstrate that healthy pediatric subjects are more prone to adapt to external force field than CP ones. The adaptation mechanism anyway is quite similar to that observed between adult stroke and control group; in fact CP children are characterized by a very lower adaptation rate as depicted by the learning index (see figure 5) and a smaller after affect in comparison to unimpaired ones. On the basis of these outcomes we shall believe that an intensive training could lead CP group to have a better performance but despite all the training exercises for such kind of pathology should be oriented towards a protocol which explicitly challenges the internal model formation: the implementation of such rehabilitation therapy might be based on a different method of observation and evaluation of subject's performance not only focused on movement accuracy but based on robust realistic computational models of motor adaptation which are able to provide insights of two complementary aspects of motor control: cortical reorganization and impedance modulation. Consistent with this hypothesis it would be possible to compare muscular co-activity in CP and unimpaired subjects emphasizing the role of impedance control in motor adaptation [54,55]. The preliminary results of the present study may have possible implications in understanding the motor recovery process in cerebral palsy, offering non-invasive and relatively simple tool to study and quantify motor control disabilities, and to drive towards a rehabilitation protocol which enhances the adaptive process in the restoration of motor functions.

## Competing interests

The authors have not competing interests as defined by the BioMed Central Publishing Group, or other interests that may influence results and discussion reported in this study.

## Authors' contributions

LM conceived, designed the experiment, performed the data analysis and drafted the manuscript. LM, FF and MP carried out the experiments; PM participated in the design of the study and manuscript composition; FF and MP participated in the coordination of the study, assisting the patients during the robot sessions; EC and PC participated in design and coordination of the research.

All authors read and approved the final manuscript.
